# Restore natural fertility of *Kit*^*w*^*/Kit*^*wv*^ mouse with nonobstructive azoospermia through gene editing on SSCs mediated by CRISPR-Cas9

**DOI:** 10.1186/s13287-019-1386-7

**Published:** 2019-08-24

**Authors:** Xiaoyu Li, Tiecheng Sun, Xiuxia Wang, Jixin Tang, Yixun Liu

**Affiliations:** 1 Institute of Nephrology, Affiliated Hospital of Guangdong Medical University, Zhanjiang, 524001 China; 2 State Key Laboratory of Stem Cell and Reproductive Biology, Institute of Zoology, Chinese Academy of Sciences, Beijing, 100101 China

**Keywords:** CRISPR-Cas9, Nonobstructive azoospermia, Spermatogonia stem cells

## Abstract

**Background:**

Male infertility is a serious social problem in modern society. Nonobstructive azoospermia (NOA) caused by germ cell gene defects is an important reason for male infertility, but effective clinical treatment for this disease has not been established.

**Methods:**

We choose *Kit*^*w*^*/Kit*^*wv*^ mouse as a research model and try to develop a new treatment strategy and “cure” its infertility. Mutant spermatogonial stem cells (SSCs) were isolated from one single unilateral testis of a 14-day-old *Kit*^*w*^*/Kit*^*wv*^ mouse and propagated in vitro. The C to T point mutation on *Kit*^*wv*^ site of these SSCs was corrected through CRISPR-Cas9-mediated homology-directed repair (HDR) in vitro. Then, the repaired SSCs were screened out, proliferated, and transplanted into the remaining testis, and complete spermatogenesis was established in the recipient testis.

**Results:**

Healthy offsprings with wild type *Kit* gene or *Kit*^*w*^ mutation were obtained through natural mating 4 months after SSC transplantation.

**Conclusion:**

In this study, we established an effective new treatment strategy for NOA caused by germ cell gene defects through a combination of SSC isolation, CRISPR-Cas9-mediated gene editing, and SSC transplantation, which brought hope for these NOA patients to restore their natural fertility.

## Background

Male infertility has become an increasingly serious social problem in modern society, and nonobstructive azoospermia (NOA), which accounts for 10% of all infertile men, is an important cause for this [1–3]. Genetic causes, including chromosomal aberrations and spermatogenesis gene mutations, are often detected on severe NOA patients [4]. Gamete-deficient NOA due to germ cells’ genetic mutations, such as *RBMY* [5], *KLHL10* [6], and *SYCP3* [7], has been incurable by assisted reproductive technologies and difficult to bear for patients [8]. Cell therapies are considered to be one of the most promising strategies to rescue this type of infertility [9].

*c-Kit* is expressed in germ cells and controls germ cell differentiation in mammalian testis [10]. Heterozygous mutant mice with *W* and *WV* mutation of this gene, the *Kit*^*w*^*/Kit*^*wv*^ mice, whose spermatogonia are considerably reduced or exhausted, have been used as an excellent animal model to develop therapeutic strategies for gamete-deficient patients due to genetic mutations by Yuan et al [9]. They isolated tail-tip fibroblasts from adult *Kit*^*w*^*/Kit*^*wv*^ mice and then derived embryonic stem cells from these cloned blastocysts obtained by somatic cell nuclear transfer. The produced mutant ESCs’ *W* site was corrected using TALEN-mediated gene editing and further differentiated into primordial germ cell-like cells in vitro and then transplanted into busulfan-treated mouse testes for spermatogenesis re-establishment. This is an encouraging strategy which can produce functional haploid cells for intracytoplasmic sperm injection (ICSI). However, since ICSI is still needed to get offsprings, the azoospermia has not been completely cured in fact. Besides, the whole treatment process is so cumbersome that it is difficult to be applied in clinical treatment for patients.

The feasibility of CRISPR-Cas9-mediated gene editing in spermatogonial stem cells (SSCs) has been reported [11, 12]. Transplanted SSCs can generate complete spermatogenesis in recipient testis [13]. If we can carry out CRISPR-Cas9-mediated gene correction on SSCs taken from NOA patients, the treatment process will be greatly simplified, the risk of tumor formation will be reduced, and even the patients are able to restore natural fertility.

Here, to explore the feasibility of this strategy, we improved our lab’s established method for isolating SSCs [14, 15]; so that we can isolate SSCs from single unilateral juvenile *Kit*^*w*^*/Kit*^*wv*^ mouse testis, CRISPR-Cas9-mediated homology-directed repair (HDR) was conducted on isolated mutant SSCs. The repaired SSCs without *WV* mutation were screened out and propagated, then transplanted into another testis of the donor mouse. Healthy offsprings were obtained through natural mating 4 months after repaired SSC were transplanted. This work provides a more convenient and more humane therapeutic strategy for NOA.

## Method

### Animals

*Kit*^*W*^*/Kit*^*WV*^ and C57BL/6 mice were obtained from the Jackson Laboratory (Bar Harbor, ME, USA). The use of mice and all pertinent surgical procedures were approved by the Animal Care and Use Committee of the Institute of Zoology, Chinese Academy of Sciences.

### SSC cultures, gene editing, and transplantation

One-step enzymatic digestion with collagenase I (GIBCO) and DNase I (AppliChem) was used for isolation of testicular cells from ~ 14 days postpartum (dpp) mice to small seminiferous tubule fragments. The fragments were plated on 100-mm dishes in mouse embryonic fibroblast (MEF) medium, one dish for one testis. Then dishes were observed under a microscope after 18 h of culture. Dishes containing germ cells were screened out, loosely attached germ cells were collected with repetitive pipetting, and these germ cells were found to migrate into the culture dish. Thereafter, cells were transferred to new freshly prepared mouse embryonic fibroblast (MEF) dish to enrich SSC cultures. SSCs were cultured in the serum-free MEMα (Invitrogen) supplemented with 25 μg/ml insulin (Sigma), 100 μg/ml transferrin (Sigma), 20 μg/ml putrescine (Sigma), 3 mg/ml BSA (ICN), 20 μg/ml ascorbic acid (Sigma), 2 mM l-glutamine (GIBCO), 55 μM 2-mercaptoethanol (Sigma), 10 mM HEPES (Sigma), 50 units/ml penicillin (Sigma), 50 μg/ml streptomycin (Sigma), 20 ng/ml human GDNF (R&D), and 5 ng/ml human bFGF (Peprotech). The enriched SSC cultures were passaged every 4 to 5 days at a dilution of 1:2 to 1:4 depending on the size of the cell clumps and the growth of the somatic cells to remove testicular somatic cells in each passage. As such, relatively pure mutant SSC clumps, which have 93% GFRa-1-positive cells and 3% c-Kit-positive cells as reported earlier (14), were routinely obtained after a total of ~ 4–5 passages.

The *WV*-sgRNA was designed and selected from http://crispor.tefor.net/, then cloned into Bbs I sites of pX458 plasmids (Addgene plasmid 48138). This plasmid and oligo DNA were transfected into SSCs with Amaxa Cell Line Nucleofector Kit L (Lonza) using Amaxa Nucleofector according to the manufacturer’s instruction.

For transplantation, SSC clumps were trypsinized into single cells and prepared in concentrations of 5 × 10^7^ cells/ml for SSC transplantation. Ten microliters cell suspension were injected into each testis of *Kit*^*W*^*/Kit*^*WV*^ mice via the efferent duct.

### Off-target effect analysis

We identified a total of 41 potential “off-target” sites by the website used in gRNA design. We examined the five highest scoring sites. DNA sequencing of PCR products amplified from these genomic sites was performed on three different passages of the successfully repaired SSC line. The five highest scoring sites and sequences of the primers for the amplification of these five off-target sites were as follows: intergenic of *Lca5* and *Sh3bgrl2* (Forward-CAGAGGCACCTGCACTT, Reverse-TGGAACCCATCCTAACG); intergenic of *Gm1815* and *Zfp353* (Forward-GTCCACATCACATCTCA, Reverse-ACATCGGACTTTACCTT); intergenic of *Kif2b* and *Gm11498* (Forward-CGCTGGCTCTATGCTTA, Reverse-CCCAAACTGGTGGTGAG); intergenic of *Epha4* and mmu-mir-6352 (Forward-GTCCTCAAAATCCACAA, Reverse-GGGCTATTAATCAACAA); intron of *Dagla* (Forward-AGCTCTTCAGATTTCCC, Reverse-TCCAGACTCTATCCCCC).

### Immunofluorescence microscopy

Testes collected from *Kit*^*W*^*/Kit*^*WV*^ mice 2 months after SSC transplantation were fixed overnight in 4% paraformaldehyde and embedded in paraffin. Tissue sections (5 μm in thickness) were obtained in a microtome and mounted on glass slides. Sections were dewaxed and rehydrated, followed by antigen retrieval in 10 mM sodium citrate buffer. After blocking with 5% BSA in PBS (wt/vol) for 1 h, the sections were incubated with primary antibody at 4 °C overnight (see Table 1). Secondary antibody conjugated with either FITC or TRITC (Jackson ImmunoResearch) at 1:200 dilution was used and incubated for an hour at room temperature. Slides were then stained with DAPI (blue) to visualize cell nuclei and mounted in prolonged anti-fade mounting medium. Fluorescence images were captured using a Zeiss LSM780 confocal microscope.

**Table 1:** Antibodies used in this study

Antibody	Vendor	Catalog no.	Host	Working dilution
Tra98	Abcam	Ab82527	Rat	1:200
GFRα-1	R&D	AF560	Goat	1:200
C-kit	Abcam	Ab5506	Rabbit	1:200
SCP3	Abcam	Ab97672	Mouse	1:200
N-cadherin	Santa Cruz	sc-7939	Rabbit	1:200
JAM-A	Thermo	36-1700	Rabbit	1:200
Lin28a	R&D	AF3757	Goat	1:200
SOX9	Abcam	ab76997	Mouse	1:200

Abcam, Cambridge, UK; Santa Cruz Biotechnology, Dallas, TX, USA; Thermo Fisher Scientific, Waltham, MA, USA; R&D Systems, Minneapolis, MN, USA

### Genotyping

We used phenotypes to determine genotypes for the *Kit*^*w*^*/Kit*^*wv*^ mouse and related strains according to the “Genotyping Protocol” provided by The Jackson Laboratory. The hybrid *Kit*^*w*^*/Kit*^*wv*^ mice are the offspring of a cross between *Kit*^*w*^*/Kit*^*+*^ (Stock No. 000692) females and *Kit*^*wv*^*/Kit*^*+*^ (Stock No. 000049) males. The *Kit*^*w*^*/Kit*^*+*^ mouse is black with a white belly spot, occasionally has a white head blaze, and tail has a white tip. The *Kit*^*wv*^*/Kit*^*+*^ mouse is gray with a light belly and white spot and a light tail. However, the *Kit*^*w*^*/Kit*^*wv*^ mouse has a white coat and black eyes. Researchers can easily distinguish these three strains of mice by their different coat color characteristics.

### Bisulfite sequencing

To confirm the DNA methylation state, bisulfite sequencing was performed using the EZ DNA methylation kit (Zymo Research) following the manufacturer’s manual. Primers for bisulfite sequencing used in this study were as follows: *H19* (Forward-TATGAGTATTTAGGAGGTAT AAGAATT, Reverse-TTTTATCAAAAACTAACATAAACCCCT); *H19 nest* (Forward-TGTAAGGAGATTATGTTTTATTTTTGG, Reverse-CCCTAACCTCATAAAACCCATAAC TAT); *Snrpn* (Forward-TATGAGTATTTAGGAGGTATAAGAATT, Reverse-AATAAACCC AAATCTAAAATATTTTAATC); *Snrpn nest* (Forward-AATTTGTGTGATGTTTGTAATTAT TTGG, Reverse-ATAAAATACACTTTCACTACTAAAATCC). To determine the methylation state of individual CpG sites, the PCR product was extracted from the agarose gel, subcloned into a pClone007 vector (TSINGKE), and then sequenced for subsequent analysis.

## Results

### Isolation of SSCs from one *Kit*^*w*^*/Kit*^*wv*^ testis

**Fig. 1:**
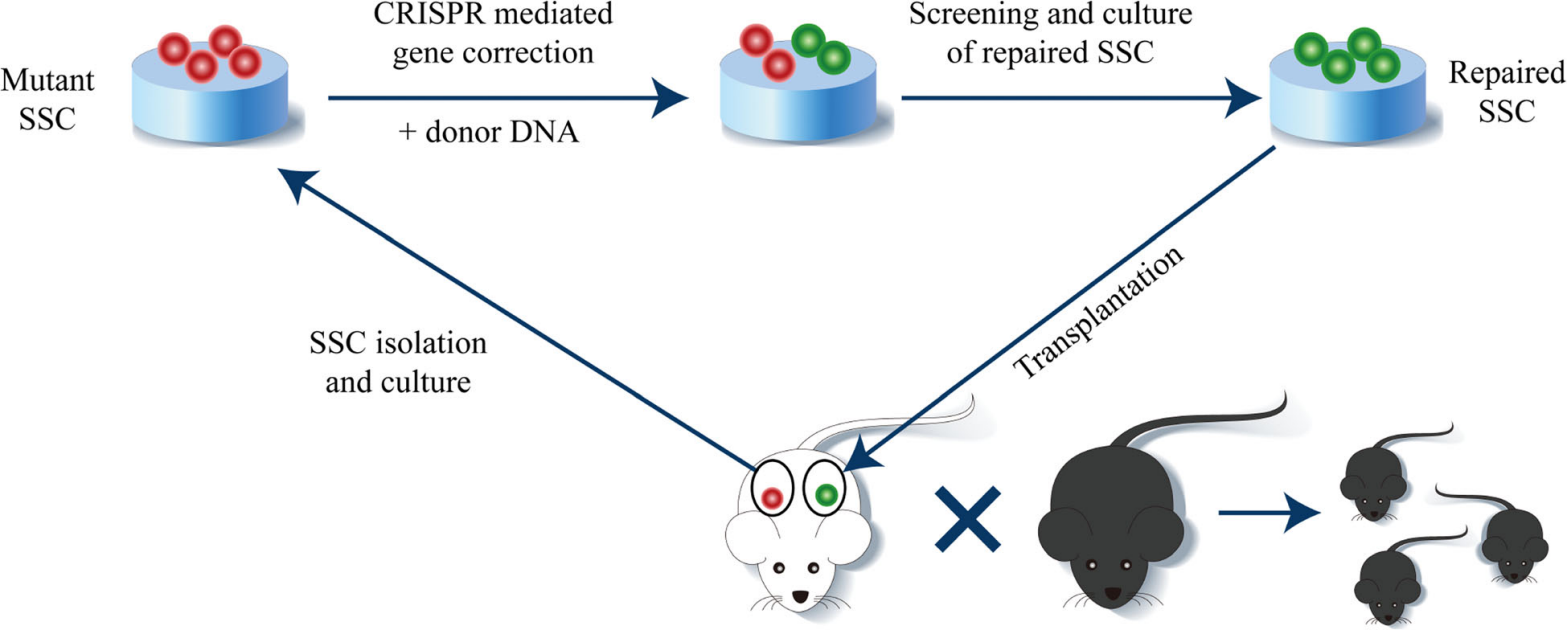
Diagram for the cure strategy of the NOA in the *Kit*^*w*^*/Kit*^*wv*^ mouse via CRISPR-Cas9-mediated gene editing in SSCs. *Kit*^*w*^*/Kit*^*wv*^ SSC cell line was established from one testis of a 14-dpp *Kit*^*w*^*/Kit*^*wv*^ mouse. Px458 plasmid with WV-sgRNA and exogenously supplied donor DNA were electroporated into *Kit*^*w*^*/Kit*^*wv*^ SSCs. GFP-positive cells, which are transfected SSCs, are enriched for further expansion and identification. SSC cell line carrying the corrected WV site without off-target mutations is selected for transplantation into the other testis of the donor mouse. The cure of the NOA was evidenced by the birth of a healthy offspring 4 months after SSC transplantation

**Fig. 2:**
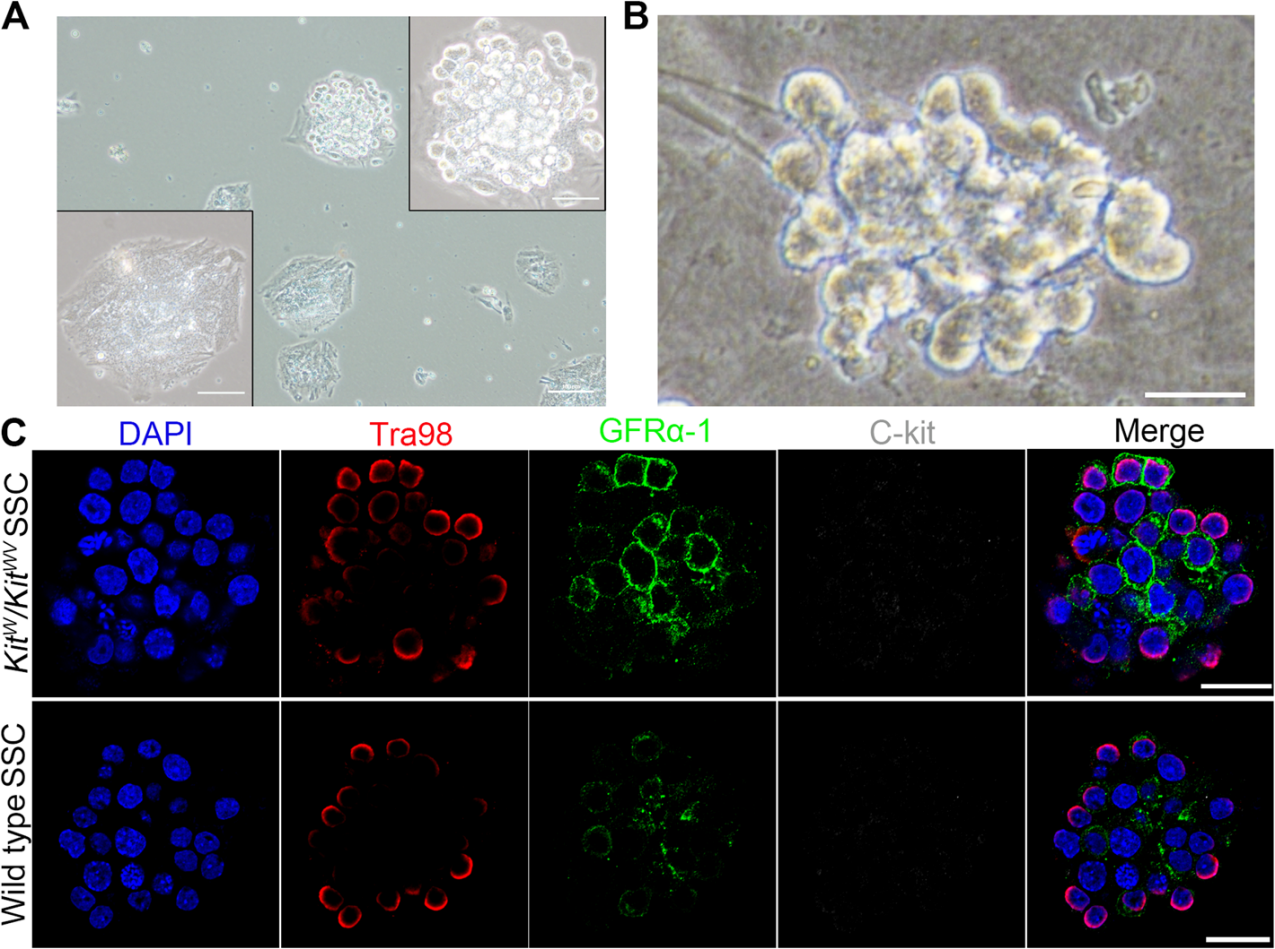
The isolating strategy and characterization of *Kit*^*w*^*/Kit*^*wv*^ SSCs. **a** All tested testes were digested separately. With the lack of germ cells, most digested testes samples were tiled in the bottom of the dish like the lower left panel after being digested into small fragments and cultured for 18 h. A small number of testes have rare tubules containing germ cells presented as the upper right panel. Testes samples containing germ cell stacks were selected for SSC enrichment. Scale bar = 100 μm in original size panels and 50 μm in magnified panels. **b** Morphology of isolated *Kit*^*w*^*/Kit*^*wv*^ SSC clump. Scale bar = 20 μm. **c** Identification of SSCs. *Kit*^*w*^*/Kit*^*wv*^ mutant SSC clumps (DAPI, for cell nuclei) were positive for germ cell marker Tra98 (red) and stem cell GFRα-1 (green), but negative for differentiated germ cell C-kit (gray), which were consistent with the wild type control SSCs shown in the bottom panel of **c**, indicating that they were undifferentiated spermatogonial stem cells. Scale bar = 20 μm

**Fig. 3:**
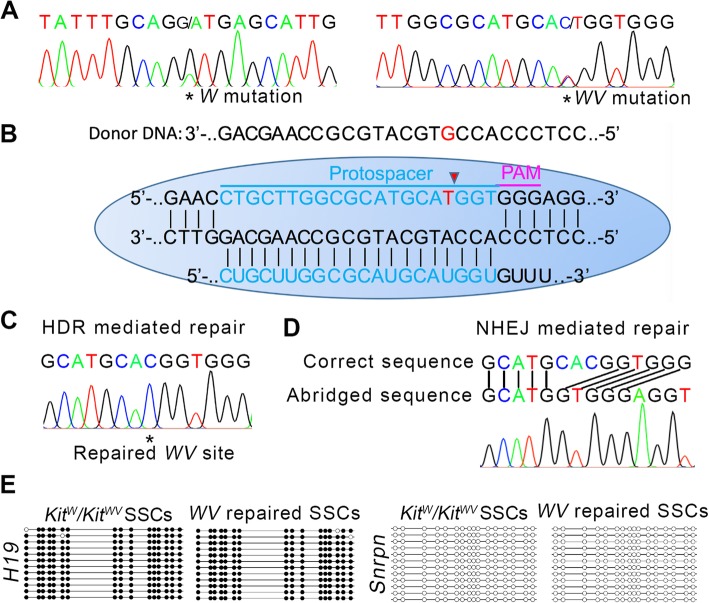
HDR-mediated correction of WV point mutation. **a** DNA sequencing of the W and WV point mutation loci in the *Kit*^*w*^*/Kit*^*wv*^ SSC cell line. Both two mutation sites showed two peaks. **b** Schematic of the Cas9-sgRNA targeting and gene correction via HDR in WV point mutation loci. Blue line labeled the sgRNA-targeting sequences and the mutant base was marked in red. **c** DNA sequencing of the WV point mutation loci in repaired *Kit*^*w*^*/Kit*^*wv*^ SSC cell line showed HDR-mediated repair gene-editing event. **d** DNA sequencing of the WV point mutation loci in typical error-repaired *Kit*^*w*^*/Kit*^*wv*^ SSC cell line showed NHEJ-mediated “CACG” deletion. **e** Methylation analysis of the DMRs of H19 and Snrpn in *Kit*^*w*^*/Kit*^*wv*^ SSCs and WV point mutation repaired SSCs. The result indicated that the repaired SSCs maintained paternal genomic imprints. Open and filled circles represent un-methylated and methylated CpG sites, respectively

The overall experiment process of this study is shown in Fig. 1. Gene-defective SSC isolation was the first step. Some of the *Kit*^*w*^*/Kit*^*wv*^ mutant testes completely lack spermatogonia, and the remaining testes contained only a few spermatogonia [16], which leads us unable to isolate SSCs as usual [15], so a donor testis screening procedure was added to the SSC isolation process. Besides, in order to ensure mouse survival after removal of unilateral testis and convenient to surgical operation, 14-dpp (day postpartum)-old mice were used. Unilateral testes taken from donor mice were digested into small seminiferous tubule fragments and respectively plated on dishes for 18 h. Most empty tubules are shown as the lower left panel of Fig. 2a, except for tubules containing spermatogonia shown as the upper right panel of Fig. 2a. Only the testis in which spermatogonia could be observed under an inverted microscope was selected for subsequent SSC separation and enrichment experiments. The obtained *Kit*^*w*^*/Kit*^*wv*^ mutant SSCs clone had normal cell morphology (Fig. 2b), and their SSC identity were confirmed by positive immunostaining of germ cell’s marker Tra98 and stem cell’s GFRα-1, and negative immunostaining of differentiated germ cell’s marker c-Kit; all these SSC identities were consistent with wild type SSCs (Fig. 2c). The presence of *W* and *WV* point mutations on isolated SSCs were confirmed by DNA sequencing analysis (Fig. 3a).

### *WV* point mutation correction

**Fig. 4:**
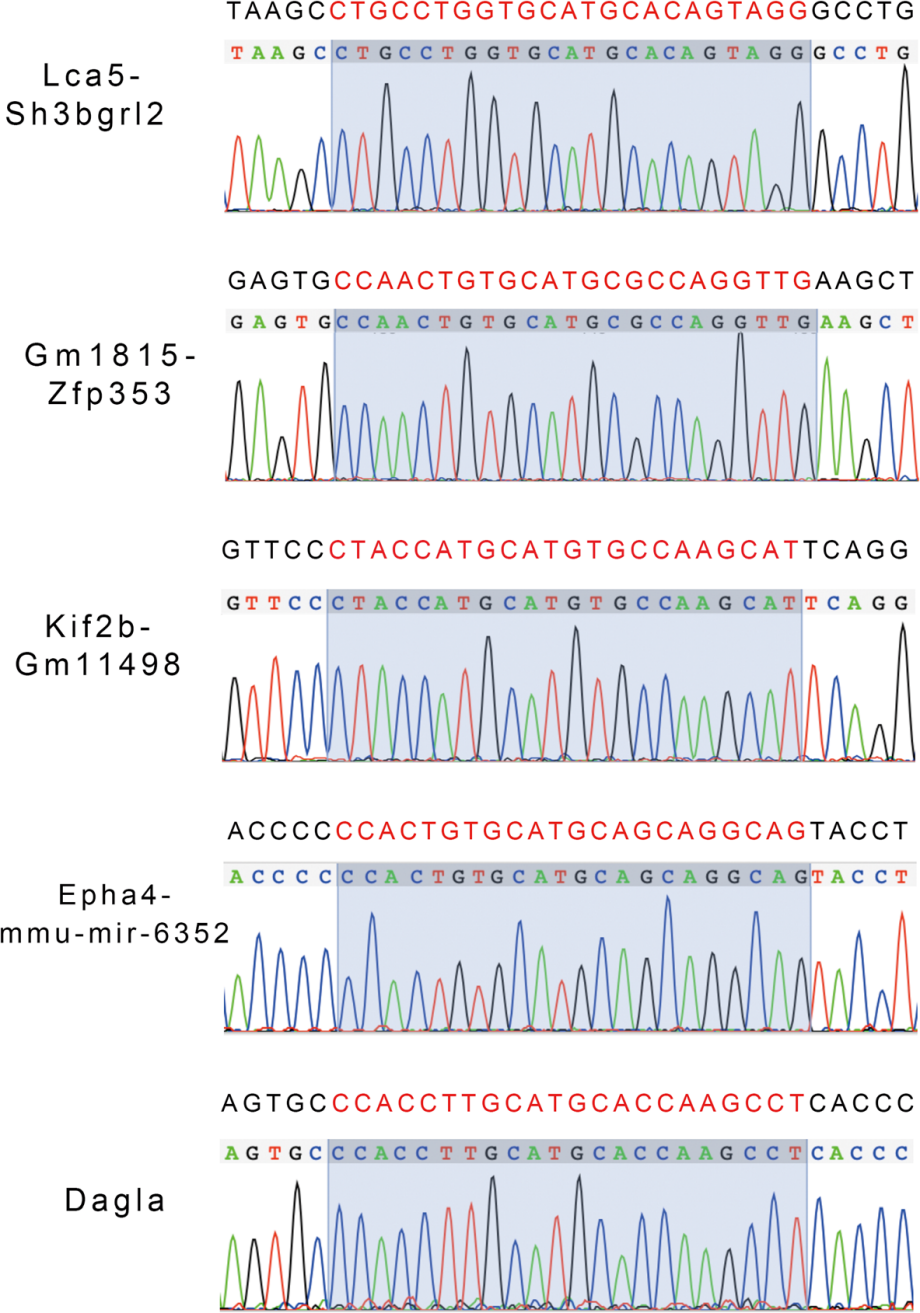
Off-target effect analysis. Sequences of the five highest scoring potential “off-target” sites were labeled with red. DNA sequencing of PCR products amplified from these genomic sites was performed. The results of DNA sequencing were consistent with corresponding genome sequences. No obvious off-target mutations were detected

Since *Kit*^*w*^*/Kit*^*+*^ and *Kit*^*wv*^*/Kit*^*+*^ mice were fertile [17], and as long as one of the two mutation sites is repaired, the *Kit*^*w*^*/Kit*^*wv*^ SSCs will restore the differentiation ability. We chose *WV* point for gene editing because there was an NGG sequence near its 3′ end. The schematic of sgRNA targeting is illustrated in Fig. 3b. We designed the single-guide RNA (sgRNA) targeting the *WV* point (termed *WV*-sgRNA) which could guide Cas9 to cut the DNA strand just at the C to T mutant site (*WV* mutation point). In order to improve the incidence of HDR, 130-bp donor DNA, whose “*WV* point” was unmutated, and pX458 plasmids expressing Cas9, GFP reporter, and *WV*-sgRNA were co-transfected into *Kit*^*w*^*/Kit*^*wv*^ SSCs. One day after electroporation, transfected SSCs were enriched by flow cytometry depending on their fluorescence characteristics and plated at low density on plates. Then single SSC colonies were picked up, and each colony was cultured in one well of the 48-well culture plate for further expansion and sequenced to screen the successfully repaired SSC lines when each clone had expanded to 2 × 10^3^ cells during passage. Twenty-one successfully expanded single SSC colonies were detected when the repaired SSC clone was screened out. Both HDR-mediated repair (one SSC clone) and nonhomologous end joining (NHEJ)-mediated deletion gene-editing events (three SSC clones) were detected after the CRISPR-Cas9 induction on the *WV* mutation site (Fig. 3c, d). Then, the SSC clone with repaired *WV* locus was screened out for further research (Fig. 3c). This SSC clone maintained paternal genomic imprints (Fig. 3e). Besides, we examined the five highest scoring potential “off-target” sites by DNA sequencing of the PCR products amplified from these genomic sites; no obvious off-target mutations were detected (Fig. 4).

### Assembly of normal blood–testis barrier (BTB) after repaired SSC transplantation and differentiation

**Fig. 5:**
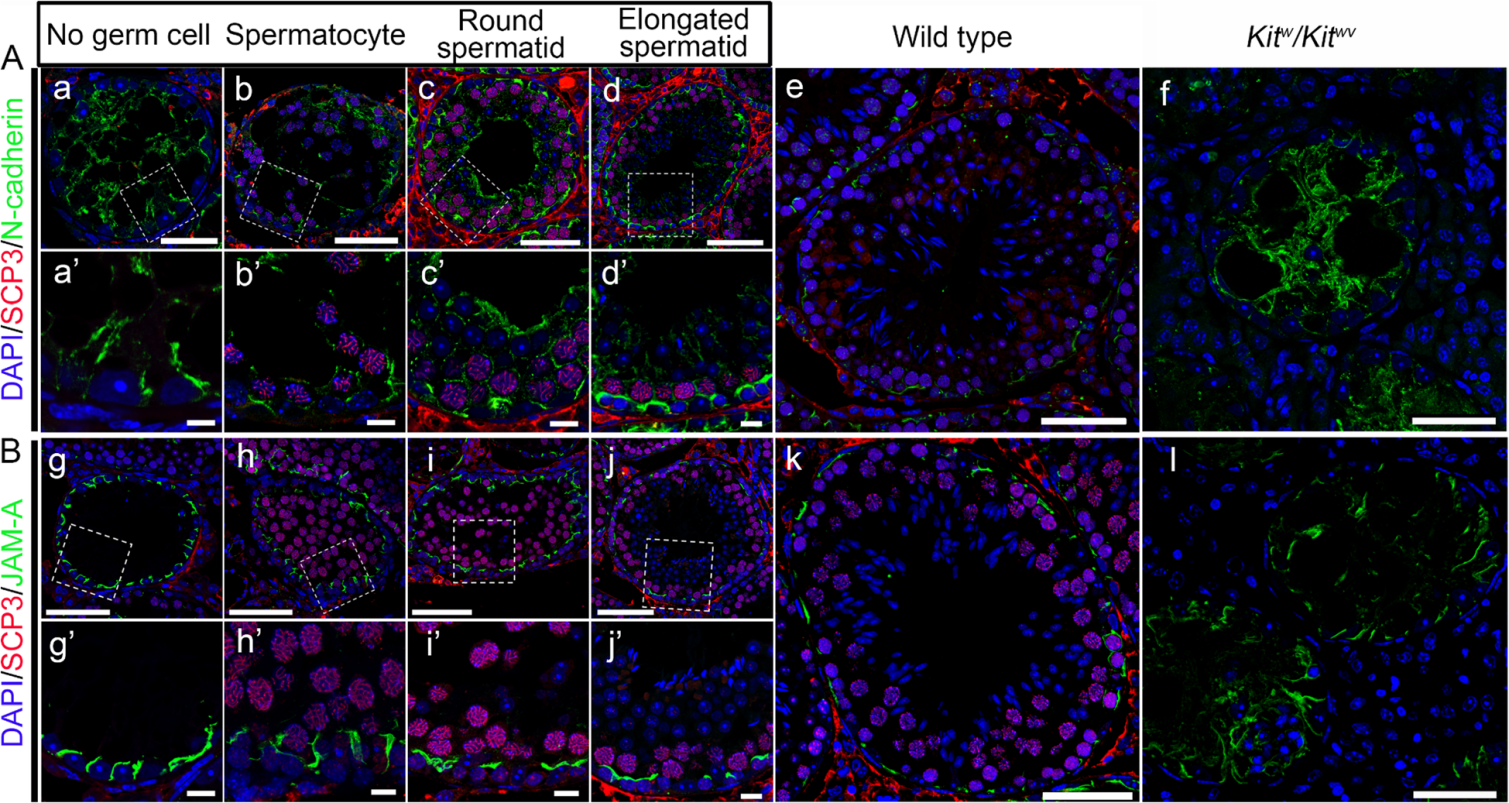
Re-establishment of BTB in the recipient testes after repaired SSC transplantation. **A**, **B** Distribution of N-cadherin and JAM-A (all in green) were found to be different in different tubules, depending on the status of spermatogenesis and the differentiation status of repaired SSCs. Abnormal distribution of these proteins in empty tubules devoid of germ cells (a and g) was obviously noted and gradually returned to normalcy as showed in normal wild type control testis (e and k) following the appearance of spermatocytes (b and h), round spermatids (c and i), and elongated spermatids (d and j). Spermatocytes were labeled by anti-SCP3 antibody (red), and round spermatids and elongated spermatids were identified by the corresponding nucleus shape (blue). There were no germ cells in panels a, a’, f, g, g’, and l. The boxed areas in a–d and g–j are magnified and shown in a’–d’ and g’–j’. Scale bar = 50 μm in a–l and 10 μm in a’–d’ and g’–j’

The BTB is one of the essential prerequisites for spermatogenesis to proceed smoothly [18]. *Kit*^*w*^*/Kit*^*wv*^ mice cannot establish a functional BTB to support meiosis due to *c-Kit* mutation usually, but the transplantation of normal SSCs can induce its assembly [15]. In order to examine if the SSCs with repaired *WV* locus could induce the assembly of functional BTB in *Kit*^*w*^*/Kit*^*wv*^ mice, the repaired SSCs were harvested and transplanted into adult *Kit*^*w*^*/Kit*^*wv*^ mice after being cultured for several passages. Then, the recipient mice were euthanized for examination by immunofluorescence analysis of typical BTB constituent proteins N-cadherin and JAM-A in the next 8 weeks so that the status of BTB assembly in cross sections of seminiferous tubules was carefully monitored. As anticipated, the functional BTB assembly was conditionality induced as normal SSCs are transplanted [15], which was directly related to the differentiation stage of germ cells within specific domains of the seminiferous epithelium. In tubules without germ cells, the distribution of N-cadherin and JAM-A was diffusely localized and extended all the way to the tubule lumen. However, they were restrictively expressed to the site in the basal region of the seminiferous epithelium where SSCs had differentiated into spermatocytes and round spermatids (Fig. 5), consistent with their location at the BTB in normal wild type control testes.

### Establishment of complete spermatogenesis and generation of healthy offsprings after repaired SSC transplantation

**Fig. 6:**
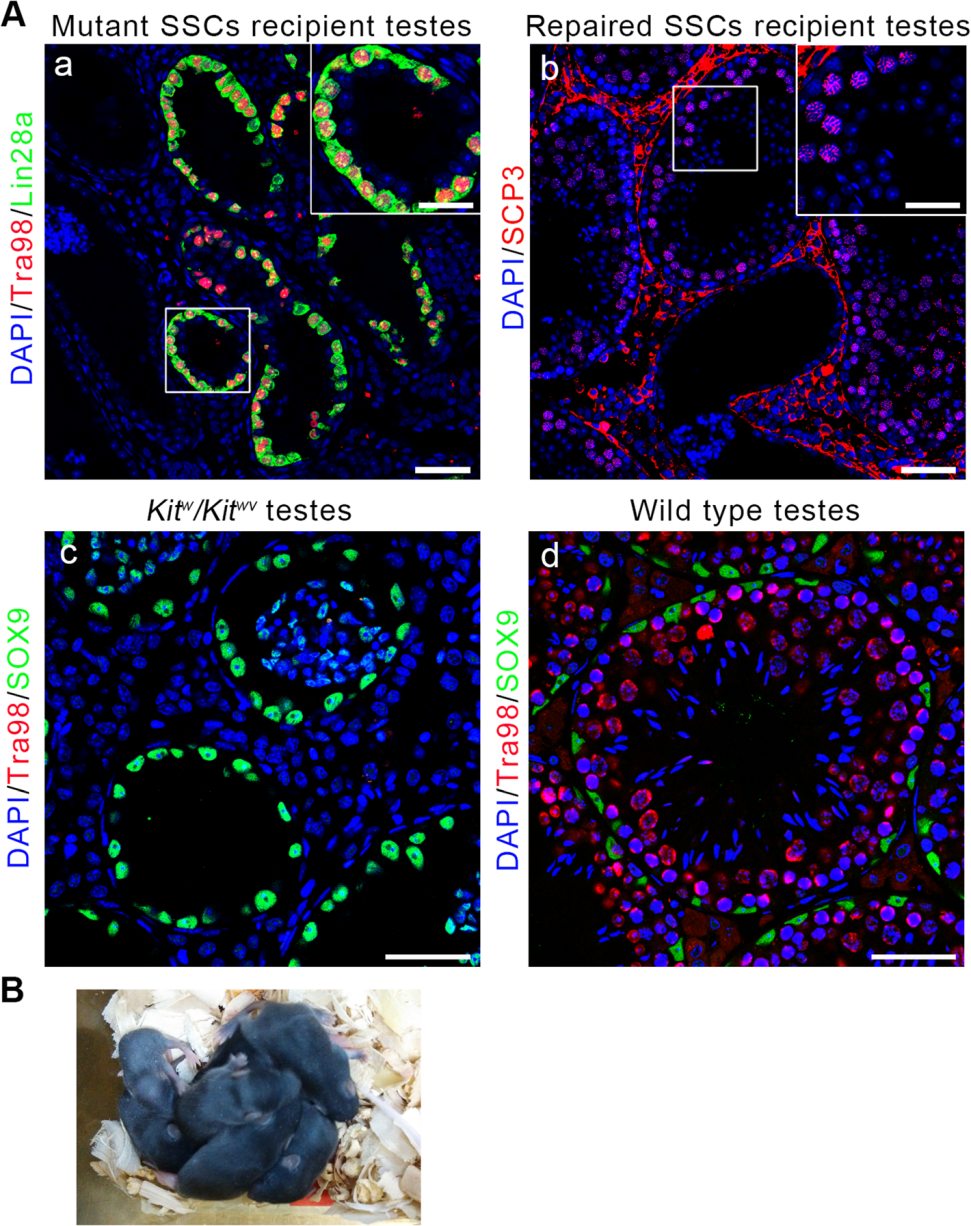
Natural fertility restoration after repaired SSC transplantation. **A** Spermatogenesis status of recipient testes 2 months after mutant or repaired SSC transplantation. The germ cells in *Kit*^*w*^*/Kit*^*wv*^ SSC recipient testes are positive for spermatogonia marker Lin28a (green) and cannot complete meiosis. In contrast, complete spermatogenesis can be found in recipient testes of repaired SSCs. The distribution pattern of germ cells in *Kit*^*w*^*/Kit*^*wv*^ testes and wild type testes were also detected by immunostaining of germ cell marker Tra98 (red) as controls. Scale bar = 50 μm in original size panels and 25 μm in magnified panels. **B** The offsprings derived from transplanted repaired SSCs

After confirming that the SSCs with repaired *WV* locus could successfully induce the assembly of functional BTB and differentiate in *Kit*^*w*^*/Kit*^*wv*^ mice, we examined if the recipient mice could establish complete spermatogenesis, and restoring fertility, the repaired SSCs were transplanted into another testis of the donor mouse and other 14 adult *Kit*^*w*^*/Kit*^*wv*^ mice. Unrepaired *Kit*^*w*^*/Kit*^*wv*^ SSCs were also transplanted as control. Two months later, three recipient mice of the repaired SSCs were sacrificed for spermatogenesis status detection. We observed that both kinds of SSCs recolonized to the basement membrane and proliferated. However, unrepaired *Kit*^*w*^*/Kit*^*wv*^ SSCs were still positive for spermatogonia marker Lin28a and could not differentiate to establish complete spermatogenesis (Fig. 6A, a). But the repaired SSCs could differentiate further and establish complete spermatogenesis in seminiferous cords (Fig. 6A, b). The distribution pattern of germ cells in the repaired SSC recipient testes was consistent with that in wild type testes (Fig. 6A, d), but significantly different from that in *Kit*^*w*^*/Kit*^*wv*^ testes lacking germ cells (Fig. 6A, c). Then, wild type and *Kit*^*w*^/*Kit*^*+*^ offsprings were obtained from the donor mouse and other 9 of 11 adult *Kit*^*w*^*/Kit*^*wv*^ recipient mice after mating with wild type C57BL/6 mice (Fig. 6B), which means the NOA of the *Kit*^*w*^*/Kit*^*wv*^ mouse was cured.

## Discussion

CRISPR-cas9-mediated gene editing has been applied on mouse and rat SSCs for the acquisition of heathy offsprings from parents with somatic cell genetic disease [11, 12]. This treatment strategy is promising and relatively easy to implement, because these “patients” had normal spermatogenesis to provide enough SSCs. However, it maybe not be suitable for NOA patients who lack germ cells. In contrast, our study has brought great hope for NOA patients with germ cell genetic disease. If their SSCs can be isolated through testicular biopsy [19] and corrected in vitro, then transplanted back to the patients’ withered testes to restart spermatogenesis, the NOA will be cured. In this study, we verified the feasibility of this treatment strategy. The establishment of complete spermatogenesis in the recipient testis after repair of SSC transplantation and generation of healthy offsprings indicated the feasibility of this strategy.

This strategy only includes SSC isolation, in vitro gene editing, and transplantation, three main steps which are greatly simplified compared to a previous report [9]. Besides, autologous transplantation is more ethical and immunotoxic reactions are removed. SSC cell lines that carry the desired gene modification without unwanted mutations can be selected easily in vitro, which brings a 100% efficiency of birth to healthy offsprings [11]. Somatic cell genetic disease also have a chance to be removed from offsprings in this treatment process. Furthermore, this strategy is more ethical and easier to be accepted for NOA patients. If it can be applied in humans, the NOA will be cured actually, which means that the patients can give birth according to their own wishes by the way of natural mating. For NOA patients without SSCs, the strategy of Yuan et al. is more applicable.

As mentioned above, this strategy is mainly aimed at the treatment of NOA caused by germ cell gene defects, especially these NOA patients whose testes have intact or partially intact stem cell niche and retained SSCs. It may not be the most suitable or economical treatment strategy for other types of NOA patients, such as azoospermic cancer survivors whose testicular stem cell niche is compromised.

Although we co-transfected a donor DNA to improve the incidence of HDR, the 4.7% (1/21) incidence rate of HDR was still low and lower than the incidence rate of NHEJ 14.2% (3/21). More efforts should be made to improve the incidence of HDR. Satisfactorily, the recipient mice had a high rate (81.8%) of successful reproduction rate after mating with wild type C57BL/6 mice, which means the repaired SSCs had a great chance to successfully restore spermatogenesis.

Researchers have made a lot of effort to reduce the off-target effect in recent years’ application of the CRISPR-cas9 system, and relatively good results have also been achieved [20, 21]. However, unpredictable and unexpected mutations after CRISPR-Cas9 editing still occur. Although no obvious off-target mutations were detected in this study, the detection scheme we used was relatively simple and not comprehensive enough. There may be off-target events that had not been detected. So stricter standards must be used if this strategy is used in a human body. More comprehensive and detailed inspection methods, such as whole genome sequencing, must be used before transplantation.

The propagation of human SSCs in vitro was reported about 10 years ago [22, 23], but there are still difficulties for other labs to replicate. So, we cannot apply this strategy on human NOA patients immediately, but we believe SSCs will play a key role in getting human offsprings without genetic disease in the near future.

## Conclusions

In this study, we established a new treatment strategy for NOA caused by germ cell gene defects through combination of SSC isolation, CRISPR-Cas9-mediated gene editing, and SSC transplantation, which brought hope for some NOA patients to restore their natural fertility. Not only to cure the NOA patients with germ cell gene defects, but may also for males who can bear normally but with other genetic disorders, be able to produce normal gametes and descendants by combining this strategy and ART technology.

## Data Availability

All analyzed data are available in the manuscript. Raw data or other materials produced in the conduct of these studies are available from the authors to qualified investigators upon request.

## Abbreviations

BTB: Blood–testis barrier
dpp: Day postpartum
HDR: Homology-directed repair
ICSI: Intracytoplasmic sperm injection
MEF: Mouse embryonic fibroblast
NHEJ: Nonhomologous end joining
NOA: Nonobstructive azoospermia
sgRNA: Single-guide RNA
SSCs: Spermatogonial stem cells
